# Admittance‐based pressure–volume loops versus gold standard cardiac magnetic resonance imaging in a porcine model of myocardial infarction

**DOI:** 10.14814/phy2.287

**Published:** 2014-04-23

**Authors:** Gerardus P. J. van Hout, Sanne J. Jansen, Johannes M. I. H. Gho, Pieter A. Doevendans, Wouter W. van Solinge, Gerard Pasterkamp, Steven A. J. Chamuleau, Imo E. Hoefer

**Affiliations:** 1 Experimental Cardiology Laboratory, University Medical Center Utrecht, Utrecht, The Netherlands; 2 Department of Cardiology, University Medical Center Utrecht, Utrecht, The Netherlands; 3 Department of Clinical Chemistry and Haematology, University Medical Center Utrecht, Utrecht, The Netherlands

**Keywords:** Admittance, myocardial infarction, PV loops

## Abstract

A novel admittance‐based pressure–volume system (AS) has recently been developed and introduced. Thus far, the new technique has been validated predominantly in small animals. In large animals it has only been compared to three‐dimensional echocardiography (3DE) where the AS showed to overestimate left ventricular (LV) volumes. To fully determine the accuracy of this device, we compared the AS with gold standard cardiac magnetic resonance imaging (CMRI) in a porcine model of chronic myocardial infarction (MI). Fourteen pigs were subjected to 90 min closed chest balloon occlusion of the left anterior descending artery. After 8 weeks of follow up, pigs were consecutively subjected to LV volume measurements by the AS, CMRI, and 3DE under general anesthesia. The AS overestimated end diastolic volume (EDV; +20.9 ± 30.6 mL, *P* = 0.024) and end systolic volume (ESV; +17.7 ± 29.4 mL, *P* = 0.042) but not ejection fraction (EF; +2.46 ± 6.16%, *P *= NS) compared to CMRI. Good correlations of EDV (*R* = 0.626, *P* = 0.017) and EF (*R* = 0.704, *P* = 0.005) between the AS and CMRI were observed. EF measured by the AS and 3DE also correlated significantly (*R* = 0.624, *P* = 0.030). After subjection of pigs to MI, the AS very moderately overestimates LV volumes and shows accurate measurements for EF compared to CMRI. This makes the AS a useful tool to determine cardiac function and dynamic changes in large animal models of cardiac disease.

## Abstract (precis)

Is the novel admittance‐based pressure–volume loop system reliable for the assessment of left ventricular volumes compared to gold standard cardiac magnetic resonance imaging in a porcine model of myocardial infarction? In the postinfarction remodeled heart, admittance‐based pressure–volume loop measurements accurately measure ejection fraction and very moderately overestimate end diastolic and end systolic volumes compared to gold standard cardiac magnetic resonance imaging, making it a very useful technique for cardiac function assessment in experimental studies.

## Introduction

Cardiac function is a main endpoint in efficacy testing of novel therapeutics in clinical and translational cardiovascular research (van der Spoel et al. 2011; Gho et al. 2013). This requires objective and reliable tools for its determination. Many current methods use left ventricular (LV) volumes as a surrogate of cardiac function. Apart from volume assessment, invasive pressure–volume (PV) measurements (PV Loops) provide researchers with more specific information on both systolic and diastolic myocardial function and dynamic changes which are not measurable with most imaging modalities (Lips et al. 2004; Burkhoff et al. 2005; Krenz 2009). However, reliable analysis of various PV‐Loop parameters depends on adequate LV volume calibration, which requires accurate LV volume assessment.

Recently, a novel method of measuring PV loops has been introduced. This admittance‐based PV Loop system (AS) differs from the classical conductance system in several ways. First, the new method does not assume a constant contribution of parallel conductance during the cardiac cycle (Kornet et al. 2001; Wei et al. 2007). Second, it differs in the way parallel conductance is separated from blood conductance making the new method less time consuming and the system easier to use. Finally, the AS applies a nonlinear relationship between conductance and volume for the conversion of blood conductance into volume (Baan et al. 1984; Wei et al. 2007; Porterfield et al. 2009). This novel method has been extensively validated in small animal models (Kottam et al. 2006, 2011; Raghavan and Kottam 2008; Clark et al. 2009; Raghavan et al. 2009; Tabima et al. 2010; Trevino et al. 2010). Only recently, the AS has been evaluated in large animal models (Kutty et al. 2013; Van Hout et al. 2013). The large animal studies demonstrated good performance, but show that the AS moderately overestimates EDV and ESV. In both studies, however, three‐dimensional echocardiography (3DE) was used as a reference standard. 3DE has been proven to be superior to the more conventional two‐dimensional echocardiography for volume measurements and is relatively inexpensive and useful for quick and noninvasive assessment of LV volumes (Dorosz et al. 2012; Greupner et al. 2012; Santos‐Gallego et al. 2012; Kawamura et al. 2013). However, 3DE has been observed to be less precise in estimating EF compared to cardiac magnetic resonance imaging (CMRI), the gold standard for volume determination (Dorosz et al. 2012; Greupner et al. 2012). 3DE is also known to consistently underestimate both EDV and ESV and this underestimation increases in diseased hearts (Mor‐Avi et al. 2008; Moceri et al. 2012). Therefore, direct comparison with CMRI is especially mandatory after postinfarction remodeling. Hence, the aim of this study was to compare the AS‐derived EDV, ESV, and EF to CMRI‐derived measurements in a closed chest porcine model of chronic myocardial infarction.

## Material and Methods

All animal experiments were approved by the institutional animal welfare committee of the UMC Utrecht and were executed conforming to the “Guide for the Care and Use of Laboratory Animals.” A total of 14 specific pathogen‐free female landrace pigs were evaluated in this study (Van Beek Lelystad, the Netherlands). Pigs (body weight 79.1 ± 5.9 kg) were subjected to myocardial infarction followed by invasive PV measurements, CMRI and 3DE at 8‐weeks follow‐up. Before any procedural intervention, animals were housed in pairs. Because of health concerns, animals were housed individually after myocardial infarction until the end of the study. Pigs were fed twice a day, water was available ad libitum.

### Infarct induction

All animals were pretreated with amiodaron for 10 days (1200 mg loading dose, 800 mg/day maintenance), clopidogrel for 3 days (75 mg/day), and acetylsalicylic acid for 1 day (320 mg loading dose, 80 mg/day maintenance). All medication was continued until the end of the study. Animals were anesthetized in their cage with an intramuscular injection of 10 mg/kg ketamine, 0.4 mg/kg midazolam, and 0.5 mg/kg atropine. Anesthesia was maintained with intravenous infusion of 0.5 mg kg^−1^ h^−1^ midazolam, 2.5 *μ*g kg^−1^ h^−1^ sufentanyl, and 0.1 mg kg^−1^ h^−1^ pancuronium and pigs were mechanically ventilated. Preoperatively, animals received a fentanyl patch (25 *μ*g/h). Arterial access was obtained by introduction of an 8F sheath into the carotid artery after surgical exposure. A coronary angiogram of the left coronary tree was acquired using an 8F JL4 guiding catheter (Boston scientific, Natick, MA). The diameter of the left anterior descending artery (LAD) was measured directly distally to the second diagonal artery. An adequately sized balloon was placed distal from the second diagonal branch and inflated for 90 min. After reperfusion and observation for approximately 2 h, the surgical wound was closed and animals were weaned from anesthesia. Animals were defibrillated in case of ventricular fibrillation (VF).

### Invasive PV‐loop measurements

Admittance‐based PV‐loop measurements were performed as recently described (Van Hout et al. 2013). In short, animals were again anesthetized according to the protocol described above 8 weeks after infarct induction. Arterial access was obtained by introduction of an 8F sheath into the carotid artery. The 7F tetra‐polar admittance catheter (7.0 VSL Pigtail/no lumen, Transonic SciSense, London, Canada) was inserted into the left ventricle through the sheath in the carotid artery under fluoroscopic guidance. The catheter measures admittance magnitude and phase in combination with pressure. It contains seven platinum electrodes dividing it into four selectable segments. The largest segment inside the LV was used for absolute volume assessment. The catheter was connected to the ADVantage system^™^ (Transonic SciSense) linked to a multichannel acquisition system (Iworx 404), required for real‐time data acquisition. A baseline scan was performed to determine the end diastolic and end systolic blood conductance required for absolute volume calculations. The external stroke volume (SV) required for volume calibration and analysis was derived from CMRI measurements. Blood resistivity was assumed to be constant in all animals (150 Ω cm). All measurements were performed during apnea. Data were offline analyzed using Iworx analysis software (Labscribe V2.0, Dover, NH).

### Cardiac magnetic resonance imaging

Immediately after PV measurements, pigs were transported to the CMRI scanner. CMRI images were obtained with a 3T CMRI scanner (Achieva TX, Philips Healthcare). Animals were placed on the CMRI table in a supine position, under continuous anesthesia. A 32‐channel receiver coil was placed over the chest. ECG‐gated steady‐state free precision cine imaging was obtained in a short‐axis and a two‐chamber long‐axis view (voxelsize acquisition = 2 × 2.1 mm, recon voxelsize = 1.25 ×1.25 mm, slice thickness = 8 mm, bandwidth = 1243 Hz, echo time = 1.62 msec, repetition time = 3.2 msec, balanced gradient echo readout = 20). Offline imaging analysis was performed in Qmass MR 7.4 enterprise solutions (Medis medical imaging systems BV, Leiden, the Netherlands).

### Three‐dimensional echocardiography

3DE was performed with a X3‐1 transducer on an iE33 ultrasound device (Philips, Eindhoven, the Netherlands) directly after CMRI as previously described (Van Hout et al. 2013). After medial sternotomy, a gel‐filled flexible sleeve was placed directly on the apex of the heart. The depth and sector size were adjusted to fit the complete ventricle. All data sets were acquired in real time using seven consecutive cardiac cycles (full volume analysis). The images were analyzed offline using QLab 10.1 (3DQ advanced, Philips, Eindhoven, the Netherlands) analysis software. Ventricle tracing was performed by semiautomatic border detection as described before (gain 50%, compression 50%, frame rate = 20–30 frames/sec, 1 dataset = 7 beats) (Soliman et al. 2007). Because of incomplete capture of the left ventricle on echocardiographic recordings, 2 of the 14 pigs were excluded from the analysis.

### Infarct size

Animals were sacrificed by exsanguinations under anesthesia. The hearts were excised and the LV was then cut into five equal slices from apex to base. Slices were incubated in 1% triphenyltetrazolium chloride (Sigma‐Aldrich Chemicals, Zwijndrecht, the Netherlands) in 37°C 0.9% NaCl for 15 min to discriminate infarct tissue from viable myocardium.

### Statistical analysis

All data are expressed as mean ± standard deviation unless stated otherwise. CMRI data and PV measurements were separately analyzed by two different researchers blinded to the outcome of the other technique. End diastolic volume (EDV), end systolic volume (ESV), and ejection fraction (EF) measured by the AS and 3DE were compared with CMRI values using a paired Student's *t*‐test. Correlations were tested using Pearson's correlation test. The limits of agreement (1.96 SD = 95% confidence interval) of the AS compared to CMRI were determined by Bland–Altman analysis. All statistical analyses were performed in SPSS statistics version 20.0 (IBM Statistics, Chicago, IL). A two‐sided *P*<0.05 was regarded statistically significant in all analyses.

## Results

**Figure 1.**
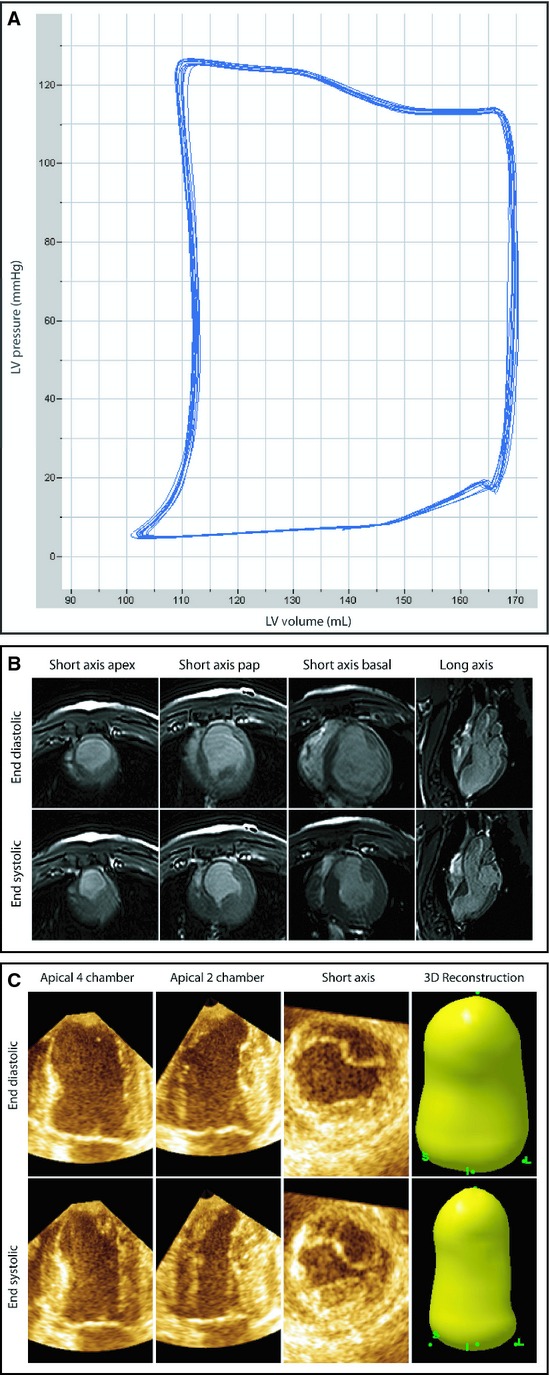
Different techniques for LV volume measurements. EDV, ESV, and EF were measured with three different modalities. (A) Representative recordings of PV loops with the AS. Ten beats were recorded during apnea. (B) Representative images of a pig subjected to CMRI measurements at the end of systole and diastole. Four different views are depicted. Short axis at the apex, papillary muscle (pap), and at a basal level combined with a long‐axis view. (C) Representative images 3DE measurements at the end of systole and diastole. Three different views are depicted. Apical four chamber, apical two chamber, and a short‐axis view, together resulting in a 3D reconstruction of the left ventricle.

At 8‐week follow‐up after MI, LV volumes were assessed in 14 pigs with both the AS and CMRI (Fig. 1A and B). Seven of the 14 animals showed at least one episode of VF. Left ventricular end systolic pressure (122 ± 27 mmHg) and end diastolic pressure (13 ± 8 mmHg) were in a physiological range during the experiment. Heart rate did not show any significant difference between AS measurement and CMRI measurements (54 ± 18 vs. 58 ± 16 bpm, *P* = NS). Moreover, heart rate correlated significantly (*R* = 0.747, *P* = 0.002) between AS‐ and CMRI‐derived measurements, indicating that both measurements were performed under a steady state.

### 3DE versus CMRI

**Figure 2.**
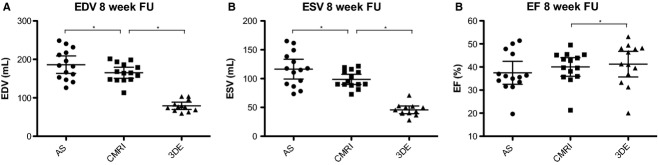
Measured LV volumes by the AS, CMRI, and 3DE in the infarcted porcine heart. LV dimensions were obtained by the three different modalities. (A) EDV 8 weeks after MI. (B) ESV 8 weeks after MI. (C) EF 8 weeks after MI. Data are presented as mean ± 95% CI. *P < 0.05. LV, left ventricular; AS, admittance‐based PV system; CMRI, cardiac magnetic resonance imaging; 3DE, three‐dimensional echocardiography.

**Figure 3.**
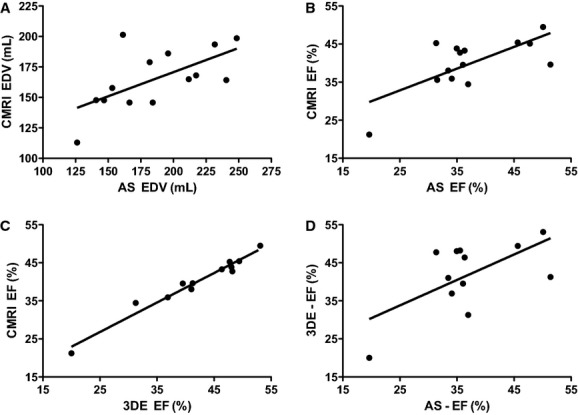
Correlation plots of LV volumes between the AS, CMRI, and 3DE in the infarcted porcine heart. (A) EDV correlates significantly between the AS and CMRI. *R* = 0.626, *P* = 0.017, *n* = 14. (B) EF correlates significantly between the AS and CMRI. *R* = 0.704, *P* = 0.005, *n* = 14. (C) EF correlates significantly between CMRI and 3DE. *R* = 0.982, *P* < 0.001, *n* = 12. (D) EF correlates significantly between 3DE and the AS. *R* = 0.624, *P* = 0.030, *n* = 12. LV, left ventricular; AS, admittance‐based PV system; CMRI, cardiac magnetic resonance imaging; 3DE, three‐dimensional echocardiography.

**Figure 4.**
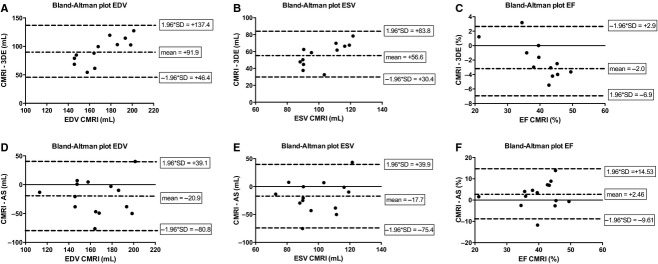
Bland–Altman plots of LV volumes in the infarcted porcine heart. Bland–Altman plots of 3DE (A–C) and the AS (D–E) with CMRI as reference standard. The green line represents the mean. The red lines represent the limits of agreement. (A) End diastolic volume. (B) End systolic volume. (C) Ejection fraction. AS, admittance‐based PV system; CMRI, cardiac magnetic resonance imaging; 3DE, three‐dimensional echocardiography.

To test the degree of LV volume underestimation by 3DE compared to CMRI, we measured LV volumes with 3DE in 12 of the 14 animals (Fig. 1C). EDV (79.1 ± 14.9 mL [3DE] vs. 165.3 ± 24.8 mL [CMRI], *P* < 0.001) and ESV (45.8 ± 10.6 mL [3DE] vs. 98.6 ± 15.2 mL [CMRI], *P* < 0.001) were underestimated by 3DE compared to CMRI as expected (Fig. 2A and B, Table 1). EF measured by MRI was slightly overestimated by 3DE (41.9 ± 9.23% [3DE] vs. 40.0 ± 7.0% [CMRI], *P* = 0.018), with an almost perfect correlation (*R* = 0.982, *P* < 0.001) (Figs. 2C, 3C, Table 2). We also determined whether LV size influenced the accuracy of the measurements by 3DE. Indeed Bland–Altman analyses revealed a trend toward a lower accuracy for EDV, ESV, and EF in the larger sized hearts (Fig. 4A–C).

**Table 1.** Assessment of LV dimensions in the infarcted porcine heart with the AS, CMRI, and 3DE.

LV parameters	AS	CMRI	3DE
EDV (mL)	186 ± 39	165 ± 25	79 ± 15
ESV (mL)	116 ± 30	99 ± 15	46 ± 11
EF (%)	37.5 ± 8.6	40.0 ± 7.0	41.9 ± 9.2

All values are presented as mean ± standard deviation; *n* = 14 for AS and CMRI measurements, *n* = 12 for 3DE measurements.

**Table 2.** Correlations between the AS, CMRI, and 3DE for EDV, ESV, and EF in the infarcted porcine heart.

LV parameters	CMRI versus AS	CMRI versus 3DE	3DE versus AS
EDV (mL)	0.626*	0.160	0.235
ESV (mL)	0.271	0.320	−0.111
EF (%)	0.704*	0.982*	0.624*

Numbers shown are the correlation coefficients (*R*).

* Significant correlation between two techniques (*P* < 0.05).

### AS versus CMRI

Next we determined whether EDV, ESV, and EF measured with the AS deviated from CMRI values. In this study, the AS significantly overestimated both EDV (186.2 ± 39.2 mL [AS] vs. 165.3 ± 24.8 mL [CMRI], *P* = 0.024) and ESV (116.4 ± 29.7 mL [AS] vs. 98.6 ± 15.2 mL [CMRI], *P* = 0.042) compared to CMRI (Fig. 2A and B, Table 1). However, EF measured by the AS was not significantly different from MRI derived EF (37.5 ± 8.6% [AS] vs. 40.0 ± 7.0% [CMRI], *P *= NS) (Fig. 2C, Table 1). Importantly, we observed a good correlation of EDV (*R* = 0.626, *P* = 0.017) and EF (*R* = 0.704, *P* = 0.005) between the AS and CMRI (Fig. 3A and B, Table 2) and a good correlation of EF between AS and 3DE (*R* = 0.624, *P* = 0.030) (Fig. 3D). Subsequently, the limits of agreement of the AS opposed to CMRI were determined by Bland–Altman analysis. Both EDV and ESV measured by the AS showed a moderate to good agreement and EF showed a good to excellent agreement with CMRI (Fig. 4). The mean percentage intermethod difference was 12% for EDV, 17% for ESV, and 6% for EF. No particular trend was detected between the accuracy of measurements and increasing values for ventricular volumes by CMRI.

### Correlation with infarct size

**Figure 5.**
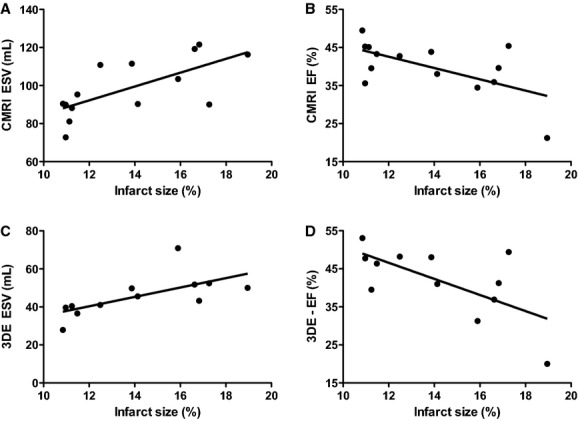
Correlation plots of LV volumes with infarct size in the infarcted porcine heart. Significant correlations of LV dimensions of CMRI and 3DE with infarct size. (A) ESV (CMRI) correlates significantly with infarct size. *R* = 0.685, *P* = 0.007, *n* = 14. (B) EF (CMRI) correlates significantly with infarct size. *R* = −0.608, *P* = 0.021, *n* = 14. (C) ESV (3DE) correlates significantly with infarct size. *R* = 0.659, *P* = 0.020, *n* = 12. (D) EF (3DE) correlates significantly with infarct size. *R* = 0.650, *P* = 0.022. CMRI, cardiac magnetic resonance imaging; 3DE, three‐dimensional echocardiography.

As LV volumes are often measured as surrogates of cardiac function, it is essential to test whether the volume measurements of the various systems reflected the actual extent of myocardial damage. Therefore, infarct size was determined at 8‐week follow‐up. Mean infarct size was 13.8 ± 2.9%. We found a good correlation of CMRI‐based ESV (*R* = 0.685, *P* = 0.007) and EF (*R* = −0.608, *P* = 0.021) with infarct size (Fig. 5A and B, Table 3). For 3DE, a similar correlation for ESV (*R* = 0.659, *P* = 0.020) and EF (*R* = −0.650, *P* = 0.022) compared to infarct size was found, indicating that both systems' measurement reflect the decay in cardiac function after myocardial infarction (Fig. 5C and D). With the AS, however, we failed to establish a significant correlation between either ESV or EF with infarct size.

**Table 3.** Correlations between ESV and EF for the AS, CMRI, and 3DE and infarct size (IS) in the infarcted porcine heart.

LV parameters	AS versus IS	CMRI versus IS	3DE versus IS
EDV (mL)	−0.146	0.239	0.365
ESV (mL)	−0.072	0.685*	0.659*
EF (%)	−0.174	−0.608*	−0.650*

Numbers shown are the correlation coefficients (*R*).

* Significant correlation between infarct size and the LV parameter (*P* < 0.05).

## Discussion

Real‐time PV loops provide researchers with more detailed information on cardiac function than most imaging modalities (Suga and Sagawa 1974; Burkhoff et al. 2005). Although some of these parameters are volume independent, many PV‐loop parameters depend on accurate determination of LV volumes. Traditional PV systems have shown to accurately estimate LV volumes but require additional calibration steps that are susceptible to technical error (Baan et al. 1984; Winter et al. 2008; Krenz 2009). The novel AS is easier to use and does not require these calibration steps that could potentially increase variability (Kottam et al. 2006).

To the best of our knowledge, this is the first study to directly compare the AS with CMRI, the gold standard for in vivo LV volume assessment. Using CMRI as a reference standard in a closed chest porcine model, the AS accurately measures EF with only modest overestimation of EDV and ESV. The AS shows small mean percentage intermethod differences and a better accuracy for EDV, ESV, and EF than 3DE in this study. Furthermore, our study shows that there is a good correlation for EDV and EF between AS and CMRI and a good correlation for EF between AS and 3DE. Both CMRI and 3DE are reflecting the extent of cardiac damage since ESV and EF of the two methods correlate well with infarct size, even though scar remodeling has already occurred after such an extensive follow‐up period and infarct size analysis has become less representative due to possible scar shrinkage and resorption (Holmes et al. 1994). Presumably this phenomenon causes the lack of correlation between infarct size and EDV measured by any method in our study.

Very recently the performance of the AS in a large animal model has also been investigated by us and others (Kutty et al. 2013; Van Hout et al. 2013). Here, an overestimation of EDV and ESV at baseline was found. This overestimation was more pronounced after MI and led to an underestimation of EF in the diseased heart compared to the reference standard.

Both large animal studies were, however, limited by the use of 3DE as the reference standard. It has recently been shown that EDV and ESV are increasingly underestimated by 3DE in more diseased left ventricles, which is in line with our findings in the present study (Fig. 4A–C) (Mor‐Avi et al. 2008; Moceri et al. 2012). The underestimation observed in our study is slightly more severe compared to clinical data. Mor‐Avi et al. found an underestimation for EDV of −67 ± 46 mL when comparing 3DE to CMRI in a heterogeneous population of patients with both healthy and failing hearts (Mor‐Avi et al. 2008). In this study, we only performed measurements in infarcted hearts, which presumably is the reason for this relatively severe underestimation. These data suggest that comparison of the AS with CMRI is warranted to fully determine the accuracy of this novel technique.

The current study has several limitations. First of all, measurements were performed under general anesthesia. Since different levels of anesthesia could influence cardiac performance, anesthesia could be a confounding factor in comparing the AS with CMRI despite a consistent heart rate. Second, during CMRI, arterial pressure was not registered. Although heart rates did not indicate a change in hemodynamic state, theoretically a difference in arterial pressure between data assessment with the different systems could occur, possibly influencing the outcome. In this study CMRI was preceded by measurements with the AS. Ideally this should be the other way round since the more invasive procedure could influence measurements with the less invasive procedure. Unfortunately, this was proven to be impossible due to logistical difficulties. Nevertheless, measurements with the AS only take up 15–20 min and the surgical intervention to reach the carotid artery is only minor without any substantial blood loss. Swine used in this study had relatively large hearts compared to averagely sized human hearts (Greupner et al. 2012). We failed to observe a trend in decreased accuracy toward the larger sized hearts, so this is unlikely to be of major influence. In this study, we did not perform measurements with a classical conductance system. Therefore, no conclusion can be drawn on the possible superiority of either one of the systems above the other. Finally, we did not take the effect of the sternotomy during 3DE on physiologic parameters into account. Animals underwent sternotomy after AS and CMRI measurements so the direct comparison of these two methods is not affected by this limitation.

## Acknowledgments

The authors have nothing to declare and gratefully acknowledge Marlijn Jansen, Joyce Visser, Martijn van Nieuwburg, Grace Croft, and Evelyn Velema for their excellent technical support and Joep van Oorschot for acquiring the MRI images.

## Conflict of Interest

None declared.
